# Genetics and Omics Analysis of Autoimmune Skin Blistering Diseases

**DOI:** 10.3389/fimmu.2019.02327

**Published:** 2019-10-15

**Authors:** Michael Olbrich, Axel Künstner, Mareike Witte, Hauke Busch, Anke Fähnrich

**Affiliations:** ^1^Medical Systems Biology, Institute of Experimental Dermatology, University of Lübeck, Lübeck, Germany; ^2^Institute of Cardiogenetics, University of Lübeck, Lübeck, Germany; ^3^Department of Dermatology, University of Lübeck, Lübeck, Germany

**Keywords:** autoimmune bullous diseases, autoantigens, HLA class II genes, systems medicine, genetics, transcriptomics

## Abstract

Autoimmune blistering diseases (AIBDs) of the skin are characterized by autoantibodies against different intra-/extracellular structures within the epidermis and at the basement membrane zone (BMZ). Binding of the antibodies to their target antigen leads to inflammation at the respective binding site and degradation of these structures, resulting in the separation of the affected skin layers. Clinically, blistering, erythema and lesions of the skin and/or mucous membranes can be observed. Based on the localization of the autoantigen, AIBDs can be divided into pemphigus (intra-epidermal blistering diseases) and pemphigoid diseases (sub-epidermal blistering diseases), respectively. Although autoantigens have been extensively characterized, the underlying causes that trigger the diseases are still poorly understood. Besides the environment, genetic factors seem to play an important role in a predisposition to AIBDs. Here, we review currently known genetic and immunological mechanisms that contribute to the pathogenesis of AIBDs. Among the most commonly encountered genetic predispositions for AIBDs are the HLA gene region, and deleterious mutations of key genes for the immune system. Particularly, HLA class II genes such as the *HLA-DR* and *HLA-DQ* alleles have been shown to be prevalent in patients. This has prompted further epidemiological studies as well as unbiased Omics approaches on the transcriptome, microbiome, and proteome level to elucidate common and individual genetic risk factors as well as the molecular pathways that lead to the pathogenesis of AIBDs.

## 1. Introduction

Autoimmune blistering diseases (AIBDs) of the skin are rare, yet potentially fatal autoimmune disorders. The autoantibodies are directed against distinct molecules expressed in the epidermis and at the dermal-epidermal junction (DEJ) of skin and/or mucous membranes. Binding of these autoantibodies ultimately leads to loss of cell-cell and cell-matrix adhesion in the skin and/or mucous membranes, which results in erosions and/or blister formation (1, 2). Antigens are presented as a cleaved fragment via the major histocompatibility complex (MHC). MHC, also known as the human leukocyte antigen (HLA) region in humans, comprises a region of 7.6 megabases (Mb) on chromosome 6p21. It is the most gene-dense region of the human genome, encoding 252 expressed loci, of which 40% are thought to play a key role in the immune system (3). The HLA region is furthermore characterized by an extraordinarily high degree of polymorphisms with more than 1000 known alleles for HLA-A and -B. The HLA class I and class II gene clusters comprise the isotypes HLA-A/-B/-C as well as *HLA-DPA1, HLA-DPB1, HLA-DQA1, HLA-DQB1, HLA-DRA*, and *HLA-DRB1*), respectively. They are involved in antigen processing and presentation, and usually show highly significant associations with autoimmune diseases, representing the strongest predisposing genetic factors (3, 4).

The development of autoimmune diseases is generally multi-factorial. Factors involved are a genetic predisposition, ethnicity, age, the environment, and gender. Autoimmune diseases show a prevalence and age of onset bias toward females (5, 6), which is particularly strong in systemic lupus erythematosus, Sjogren's syndrome, and autoimmune thyroiditis with females representing over 85% of all cases. In rheumatoid arthritis (RA) and multiple sclerosis, 60–75% of patients are female (5, 7). Sex-specific immune responses were also observed in mice. Female mice produced more antibodies and showed a stronger T cell activation than male mice after immunization (7–9). Yet, similar approaches in humans, in which responses to vaccination were analyzed, showed mixed results, with either no differences between males and females or an increased antibody response in females (9). It was found that females have a higher absolute number of CD4+ T lymphocytes than men (10) and produce more Th1 cytokines after vaccination. While these observations are still not completely understood, recent studies suggest that differences in the sex hormone composition, like progesterone and testosterone, may explain the differences in the immune mechanisms and autoimmune disease prevalence in females (5, 9, 11). Sex steroids may directly influence the immune system and affect components of antigen presentation, lymphocyte activation, cytokine gene expression, and/or homing of immune cells. They may also have indirect effects on corticosterone-cortisol concentrations, which are higher in females than in males. Additionally, glucocorticoids suppress the production of sex hormones and their mechanism of action in tissues (5, 11).

So far, more than 80 autoimmune diseases are known, ranging from familiar types like RA to rare forms like myasthenia gravis. Likewise, AIBDs can be subdivided into two major groups: pemphigus and pemphigoid diseases, based on the autoantigen localization. In the following sections we provide a brief description on the different AIBDs, together with their clinical manifestation and known genetic predispositions. An overview over the most common AIBDs is provided in Table 1.

**Table 1:** Autoimmune skin blistering diseases: summary of targeted antigens, produced antibodies and associated genetics of common phemphigus and pemphigoid disease.

	**Name**	**Antigen**	**Antibody**	**Genes**	**Cormorbidity**	**Source**
	**Pemphigus**					
PV	Pemphigus Vulgaris	Dsg3, Dsg1	IgG, IgA, IgM, C3	*C3, HLA-DRA, TNF, IL6, IL6R, IL10, IL10RA, IL10RB, TAP2, GP9, DSG1-4, DUSP5, ST18, CD86, ANXA9, DSP, PPL, DST, DSC3, CDH1*	Hypothyroidism, IBD, T1DM, SLE, Sjörgren's Syndrome, Alopecia Areata, AITD, Juvenile Rheumatoid Arthritis, Peridontitis	(12–16)
PF	Pemphigus Foliaceus	Dsg1	IgG4	*DSG1, FOXP3, PPL, EVPL, DST, HLA-DRB1, CTLA4*		(12, 14, 16)
PH	Pemphigus Herpetiformis	Dsg1 a/o Dsg3, Dsc1, Dsc3	IgG, C3	*DSG1, DSG3, DSC1, DSC3, DST, C3*	Psoriasis thyroid disease, SLE, HIV, lung cancer, esophageal carcinoma, prostatic cancer, cutaneous angiosarcoma	(16–20)
PNP	Paraneoplastic Pemphigus	BP180, BP230, Dsg1-3, desmoplakin1;2, envoplakin, pectin, periplakin, A2ML1, epiplakin	IgG, C3	*COL17A1, DST, DSG1, DSG2, DSG3, DSP, EVPL, PLEC, PPL, EPPK1, A2ML1, C3, DSC1, DSC2, HLA-DRB1*	Carcinoma, Thymoma, Sarcoma, Non-Hodgkin lymphoma, Chroniclymphcytic leukemia, Castlemandisease	(12, 16, 20, 21)
AP	IgA Pemphigus	SPD type: Dsc1 IEN type: Dsg1 (1 case), unknown	IgA, IgG, C3	*DSC1, DSG1, DSG3, C3*	IgA gammopathy to myeloma, cancer, CD, Gluten-sensitive enteropathy (single case)	(16, 20, 22)
PE	Pemphigus Erthematosus	Dsg1, Dsg3	ANAs: Ro/La/Sm, IgG	*DSP, DSG1, DSG3*		(16, 23)
	**Pemphigoid**					
BP	Bullous Pemphigoid	BP180 NC16A, BP230	IgG, C3, C5, IgA, IgE	*COL17A1, DST, C3, C5, HLA-DRB1, HLA-DQB1, IL3, IL3RA, IL4, IL4R, IL5, IL5RA, IL6, IL6R, IL7, IL7R, IL8, IL10, IL10RA, IL10RB, IL15, IL15RA, TNF, CCL2, CCL5, CCL11, CCL13, CCL18, FIGF, ICAM1, DSP, DSG1, PPL, EVPL, ITGB4, ITGA6*	Diabetes mellitus	(16, 24–27)
PG	Pemphigoid Gestationis	BP180 NC16A, BP230	IgG	*COL17A1, DST, HLA-DRA*	Pregnancy	(12, 16, 28)
EBA	Epidermolysis Bullosa Acquisita	COL7	IgG, IgA, IgM, IgE, C3(a) (1/3 of patients), C5(a)	*COL7A1, C3, C5, HLA-DRA, FCGR1A, SYK, LTB4R, LTB4R2, FLII, IL6, IL6R, RORA, PIK3CB, PIK3CG*	IBD, UC, CD, Myeloma, SLE amyloidosis, thyroiditis, multiple endocrinopathy syndrome, RA, pulmonary fibrosis, chronic lymphocytic leukemia, thyoma, diabetes	(12, 29–33)
ALP	Anti-Laminin γ1 / p200 Pemphigoid	Laminin γ1	IgG	*LAMC1*		(12, 16, 34)
LABD	Linear IgA-Dermatosis	Ladinin 1, COL7	IgA	*COL17A1, DST, LAD1, CD79A, ITGB4*		(12)
MMP	Mucus Membrane Pemphigoid	BP180, BP230, Laminin 332 (laminin-5), Laminin 311 (laminin-6), α6 and β4 Integrin	IgG, IgA, C3	*COL17A1, DST, LA-MA3, LAMB3, LAMC2, LAMB1, LAMC1, ITGB4, ITGA6, C3, CD79A, SERPINH1, CFP, TNFRSF1B*	Peridontitis	(12, 16, 35)

*SPD type, Subcorneal Pustular Dermatosis type; IEN type, Intraepidermal Neutrophilic type; ANAs, Antinuclear Antibodies; IBD, Inflammatory Bowel Disease; T1DM, Type I Diabetes Mellitus; SLE, Systemic Lupus Erythematosus; AITD, Autoimmune Thyroid Disease; HIV, Human Immunodeficiency Virus; CD, Crohn's Disease; UC, Ulcerative Colitis; RA, Rheumatoid Arthritis*.

### 1.1. Pemphigus Diseases

**Figure 1:**
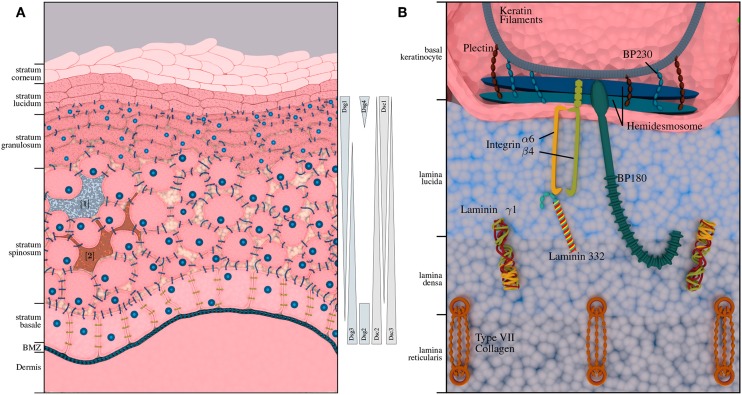
Structural composition of human epidermis and basement membrane zone (BMZ). **(A)** Shows the composition of the human skin, including melanocytes [1] (3) and immunocompetent Langerhans cells [2] (4). The approximate distribution of pemphigus antigens within the layers of the epidermis is depicted on the right-hand side. While desmogleins 1 and 4 and desmocollin 1 are expressed in the upper layers of the epidermis; desmogleins 2 and 3 as well as desmocollin 2 and 3 are expressed in the lower layers of the epidermis. **(B)** Depicts the BMZ with its cellular adhesion proteins, connecting epidermis and dermis, which are the main autoantigens in pemphigoid diseases.

Pemphigus diseases are characterized by intraepidermal autoantibody binding. The two major pemphigus types are pemphigus vulgaris (PV) and pemphigus foliaceus (PF), accounting for 70% and 15–20% of all pemphigus cases, respectively. Rarer forms of pemphigus diseases include pemephigus herpetiformis (PH), paraneoplastic pemphigus (PNP) and IgA pemphigus (AP). The incidence of pemphigus diseases is population-dependent (36) and ranges between 0.7 and 5 per million/year. It is highest in Central Europe and the United States with an estimated range for new cases between 1 and 7 per million/year. PV is between 4- and 10-fold more common among the Jewish population as compared to other Caucasian populations (37). In contrast, PF is not particularly prevalent among the Jewish population, but endemic PF-variants have been described in South America and Tunisia (38). Another endemic form of PF, namely fogo selvagem, occurs in rural areas of Brazil with a prevalence of 3.4% on certain Amerindian link reservations and an incidence of 1–4 cases per 1,200 persons per year (38). Depending on the subtype, pemphigus antibodies are mostly directed against desmoglein 3 (Dsg3) and desmoglein 1 (Dsg1). In some cases, antibodies against other antigens, such as desmocollins (Dsc) and plakins can be found. The abundance of transmembrane glycoproteins varies with the different skin layers (cf. Figure 1A); Dsg1 is predominantly expressed in the upper layers, while Dsg3 is expressed in the lower layers of the epidermis (39). Degradation of these structures leads to a loss of cell-cell adhesion and formation of intra-epidermal blisters. Accordingly, this AIBD subtype is also referred to as intra-epidermal blistering disorders.

#### 1.1.1. Pemphigus Vulgaris

PV is a pemphigus disease with autoantibodies against Dsg3 and, in some cases, additionally against Dsg1. Due to the expression of Dsg3 in the lower layers of the epidermis and in the epithelium, PV mainly affects mucous membranes. Skin involvement is determined by the presence of Dsg1 autoantibodies: In mucocutaneus PV, both Dsg1 and Dsg3 autoantibodies can be observed (40).

The age of onset is around 50–60 years. However, early and even childhood cases of PV have been reported. PV predominantly affects females (female-to-male ratio of 1.5:1) (41). Additionally, the outcome of PV may be worse in females, as a recent analysis showed that the HLA alleles DRB1*04:02 and DQB1*03:02 were associated with severe PV, and DQB1*03:02 were found more frequently in female as compared to male patients (42, 43).

**Search strategy and selection criteria**

Literature: We searched the public literature databases PubMed, ResearchGate and Google Scholar, using the terms *AIBD, autoimmune blistering disorders of the skin, pemphigus* and *pemphigoid*. The search covered articles in English published between Jan 1, 2015, and March 31, 2019. Further publications, beyond the denoted selection criteria, were selected from the reference lists of the retrieved articles.

Data repositories: The NCBI databases, particularly Gene Expression Omnibus (GEO), and European Nucleotide Archive (ENA) were searched for high-throughput data studies of the outlined phenotypes.

So far, the genetic association between PV and HLA class II genes remains the strongest and the most reported. While some of the HLA types are more population specific, others are associated with PV across different ethnicities. Several studies have found associations between PV and HLA class I alleles including *HLA-A3, HLA-A26*, and *HLA-B60* in the Han Chinese population; *HLA-B38, HLA-C12, HLA-B57*, and *HLA-C15* in the Brazilian population; *HLA-A10*, and *HLA-B15* in the Japanese population; *HLA-B35* and *HLA-B44* in the Turkish population; *HLA-B38* in the Jewish and Spanish population; and *HLA-B4402, HLA-C0401*, and *HLA-C1502* in the Iranian population (13, 44–46), respectively. Population studies have shown an association between certain class II alleles and PV in different ethnic groups. For example, HLA-DRB1*0402 is associated with PV in over 90% of Ashkenazi Jews, and HLA-DQB1*0503 is associated in non-Jewish populations. Likewise, HLA-DRB1*1404 is the most important risk factor in an Indo-Asian population. The two most common PV-associated alleles are HLA-DQB1*0503 and HAL-DRB1*0402, both of which were found to be associated with the disease in the Spanish, French, Italian, Slovak, North American and Brazilian population (47). In addition, several studies have shown an association between PV and non-classic HLA class Ib alleles (*HLA-E, HLA-F*, and *HLA-G*). *HLA-G* polymorphisms were found to have a significant association with Jewish PV patients, while *HLA-E*, previously demonstrated to play a role in multiple autoimmune conditions, was found in association with Caucasian and Ashkenazi Jewish patients and was suggested to be involved in the disruption of immune tolerance in PV (44–46, 48).

#### 1.1.2. Pemphigus Foliaceus

In PF, the autoantibodies are directed against Dsg1, while Dsg3 antibodies cannot be detected. Thus, PF affects only the skin, while mucosal lesions are completely absent (39, 49). Skin lesions are also more superficial than in PV, with desquamation/scaling rather than erosions involving the deeper skin layers. The average age of onset for sporadic PF is between 50 and 60 years with no reported gender bias. There are endemic forms of PF in Tunisia, Brazil, Peru and Colombia, which diverge in rate of incidence and observed sexual predisposition. For example, the Brazilian and Tunisian forms of PF present with higher incident rates (50, 51). A male prevalence is observed in Colombian PF, where about 95% of the cases are reported in males (52). The endemic subtypes in particular, indicate the role of environmental factors in their pathogenesis. The hotspot regions for endemic PF are characterized by poor living standards and hygienic conditions, low age of onset (around 20 years), and a seasonally varying incidence rate, which is highest at the end of the rainy season and lowest in dry summers.

Previous studies have shown that the DSG1 gene is polymorphic and that a coding synonymous T/C single nucleotide polymorphism at position 809 is associated with PF. To determine whether the disease occurs due to complex genetic interactions, it was tested whether MHC class II genes and DSG1 polymorphisms contribute to PF sensitivity. An analysis performed in 31 PF patients and 84 healthy controls first confirmed the previously reported common DRB1*04 and DRB1*14 genetic background in PF and individualized DRB1*0102, DRB1*0402 and DRB1*0406, and DRB1*1404 as susceptibility MHC class II alleles in French Caucasian PF patients (47, 53).

#### 1.1.3. Pemphigus Herpetiformis

Pemphigus herpetiformis (PH), also known as mixed bullous disease, eosinophilic spongiosis in pemphigus or acantholytic herpetiform dermatitis, is considered a clinical variant of pemphigus that combines the clinical features of dermatitis herpetiformis with the immunopathologic features of pemphigus. It accounts for 6–7.3% of all pemphigus patients. Clinically, PH is characterized by erythematous, itchy blisters and hive like swellings on several areas of the body. In contrast to PV and PF, the characteristic intense inflammation may not be associated with acantholysis (54, 55). Even though the phenotype closely resembles the features presented in dermatitis herpetiformis, its immunologic features conform to pemphigus (19). Autoantibodies in PH mainly target Dsg1 and, less commonly, Dsg3. Recently, several cases of PH without anti-Dsg1 or anti-Dsg3 autoantibodies have been reported with reactivity against other antigens such as desmocollin (Dsc) (56). It is currently unclear why the same autoantibodies result in a different clinical representation for PH and PF/PV. One explanation could be preferential binding to different epitopes on the same antigen molecule.

#### 1.1.4. Paraneoplastic Pemphigus

Paraneoplastic pemphigus (PNP) is an AIBD that may be accompanied by both malignant and benign neoplasms which are often hematologic and lymphomatoid. The most frequently associated malignancies are chronic lymphocytic leukemia, B cell lymphoma, Castleman's disease, thymoma, and Waldenstrom's macroglobulinemia (21). Autoantibodies in paraneoplastic pemphigus typically target Dsg3 and proteins of the plakin family, including periplakin, envoplakin, plectin, desmoplakin 1 and 2, BP230, and the protease inhibitor alpha-2-macroglobulin-like-1 (57). The average age of onset for PNP is 51 years with no reported gender preference. Due to the association with neoplasms, PNP is hypothesized to be a side-effect of an antitumor response that cross-reacts with epithelial cells, either because the tumor is comprised of epithelial tissue or anomalously produces desmosome-like junctions (21). However, it should be considered that other pemphigus diseases may also be associated with malignancy (58).

#### 1.1.5. IgA Pemphigus

IgA pemphigus is characterized by IgA autoantibodies against desmosomal and non-desmosomal keratinocyte cell surface components. The two major types of IgA pemphigus are subcorneal pustular dermatosis (SPD) and intraepidermal neutrophilic IgA dermatosis (IEN). The autoantigen of the SPD type was identified as Dsc1, while the antigen of the IEN type is variable (59, 60). However, in some reported cases of IEN type IgA pemphigus, IgA autoantibodies reacted with Dsg1 or Dsg3 (20, 59, 60). IgA pemphigus may be associated with monoclonal IgA gammopathy, multiple myeloma, HIV infection, Sjogren's syndrome, RA, and Crohn's disease. It is still unclear whether these diseases precede or follow IgA pemphigus. As one of the rarest AIBDs, the knowledge on IgA pemphigus is limited. No evident gender prevalence has been reported so far and the disorder may affect all age groups (22).

#### 1.1.6. Pemphigus Erythematosus

Pemphigus erythematosus, also known as Senear-Usher syndrome, was originally described as a variant of pemphigus with features of lupus erythematosus but is today regarded as a localized form of PF and is considered an AIBD in it's own right. Autoantibodies target Dsg1, but may further target Ro, La, Sm, and double-stranded DNA antigens (23, 61). Clinically, blistering coincides with a seborrheic erythematous rash resembling the rash associated with lupus (61).

### 1.2. Pemphigoid Diseases

Pemphigoid diseases are characterized by autoantibodies against connective molecules at the DEJ, which is shown in Figure 1B. Binding of the autoantibodies leads to inflammation at the DEJ and degradation of the anchoring filaments and fibrils, resulting in sub-epidermal blistering. Accordingly, this group is also referred to as sub-epidermal blistering disorders. The most common pemphigoid diseases include bullous pemphigoid (BP), and mucous membrane pemphigoid (MMP). Other, less common types include pemphigoid gestationis (PG), epidermolysis bullosa acquisita (EBA), linear IgA dermatosis (LABD), and anti-laminin γ1 / p200 pemphigoid.

#### 1.2.1. Bullous Pemphigoid

Bullous pemphigoid (BP) is the most common pemphigoid disease in Central Europe, with an incidence rate of about 10–20 per million/year. It is characterized by sub-epidermal blistering accompanied by inflammatory cell infiltration in the upper dermis (62). There are two major target antigens in BP patients: Bullous Pemphigoid Antigen 2 (*BPAG2* also known as *BP180* or type XVII collagen), and the Bullous Pemphigoid Antigen 1 (*BPAG1*, also known as *BP230*), a cytoplasmic plakin protein family member that links the hemidesmosome to the keratin of intermediate filaments. *BP180* is a transmembrane glycoprotein that extends from the intracellular domain of basal keratinocytes to the lamina densa. The immunodominant region of BP180 is the noncollagenous domain 16A (NC16A). *BP230* is a 230-kDa protein with an intracellular component associated with the hemidesmosome plate belonging to the family of plakin proteins [Figure 1B; (63, 64)]. The major immunoglobulin class in BP is IgG. It has been shown however, that some patients also develop anti-BP180 IgA and IgE autoantibodies. In fact, most of the BP sera contain both IgG and IgA autoantibodies to *BP180* (65). Autoreactive CD4+ T lymphocytes recognize unique epitopes within the extracellular region of *BP180*. *BP180*-reactive Th cells and IgG autoantibodies recognize similar or identical epitopes clustered in distinct regions of the *BP180* ectodomain and *BP230*. Many polymorphisms of HLA-II class alleles have been identified in patients with BP in several populations, especially HLA-DQ alleles. These polymorphic HLA class II alleles are likely to occur due to changes in the active binding site on the HLA molecules for binding autoantigenic peptides. A common HLA class II allele, HLA-DQB1*0301, is positively associated with BP in multiple populations and also appears to be associated with distinct clinical pemphigoid variants. Computer-based models demonstrate that the HLA-DQB1*0301 allele is capable of binding to multiple T cell epitopes within *BP230* and *BP180* for BP and α6 integrin, and β4 integrin for MMP. Binding leads to the activation of antigen specific T cells interacting with B cells through CD40/CD40L interaction, to produce four distinct anti-BMZ antibodies with different specificities. These antibodies bind to their specific target antigen resulting in the production of subepidermal blisters. In addition, the activation of BP180-autoreactive T cells from a cohort of BP patients with HLA-DQB1*03:01, was found to be restricted by this BP-associated HLA class II allele (46, 66–70).

#### 1.2.2. Pemphigoid Gestationis

Pemphigoid gestationis (PG) is a rare dermatosis that occurs during pregnancy with a reported incidence rate between 0.5 and 2 per million/year (71–73). It usually affects pregnant women during the third trimenon and, less commonly, during the second trimenon or post-partum period (28, 74, 75). When occurring during the first pregnancy, the disease reoccurs in following pregnancies in 90% of the cases. PG persists and converts to BP in less than 5% of patients. In contrast to BP, blisters are infrequent and usually small in size with predominating urticarial erythema that first affects the periumbilical region. Autoantibodies are mainly directed against *BP180* NC16A and in 10% of the cases against *BP230*. The main IgG subclasses are IgG1 and IgG3 and a strong association with maternal *HLA-DR3* and *HLA-DR4* exists (74, 75).

#### 1.2.3. Epidermolysis Bullosa Acquisita

In epidermolysis bullosa acquisita (EBA), the autoantibodies are directed against type VII collagen, an anchoring fibril at the BMZ. Both skin and mucous membranes can be affected by EBA, albeit the latter to a lesser extent. The EBA incidence rate has been reported to be between 0.2 and 0.5 new cases per million/year (76). Clinically, mechanobullous (classical) EBA and inflammatory EBA can be distinguished. A characterizing feature of the classical variant is fragility of the skin that usually affects the trauma-prone areas, such as the extensor side of joints. The inflammatory subtype can resemble other AIBDs in clinical presentation as well as in serologic and histologic findings. Common to both types is the scarring and formation of milia. The scarring is particularly problematic in mucous membranes as it reduces the tissue function, even after successful suppression of the disorder (73). Inflammatory bowel disease (IBD) has been reported in 20% of the EBA patients. A link between IBD and EBA is strengthened by the presence of type VII collagen in the colon and the finding of its respective autoantibodies in IBD patients (33). EBA has been associated with environmental factors and genetically with the MHC locus *HLA-DR2* notably the DRB1*15:03 allele in patients of African descent (73).

#### 1.2.4. Anti-p200/Anti-Laminin γ1 Pemphigoid

Anti-p200 pemphigoid is a pemphigoid disease with IgG autoantibodies against a 200 kDa protein at the DEJ (77), while IgA reactivity has also been reported (78). In 90% of anti-p200 pemphigoid cases, laminin γ1 is the target antigen (34). Therefore, anti-p200 pemphigoid is also known as anti-laminin γ1 pemphigoid. However, because reactivity with laminin γ1 cannot be demonstrated in all patients with anti-p200 pemphigoid, it is recommended to restrict the term “anti-laminin γ1 pemphigoid” to those patients with reactivity against laminin γ1 (40). The clinical presentation is highly variable, often resembling BP or the inflammatory variant of EBA, and lesions heal without scarring or milia formation (77). Mucous membranes may be affected (79) and one third of the patients present with psoriasis as a co-morbidity (73). Due to variable clinical representation, histopathology, serological, and direct immunofluorescence microscopy findings, the diagnosis requires specialized assays. Therefore, anti-p200/ anti-laminin γ1 pemphigoid might be underdiagnosed (73).

#### 1.2.5. Linear IgA Bullous Dermatosis

Linear IgA bullous dermatosis (LABD) is a pemphigoid disease with an incidence rate of about 0.2–2.3 per million/year. The autoantibodies (mainly IgA) are directed against antigens with various molecular weights, including 97-, 120-, 180-, 200-, 230-, 280-, 285-, and 290-kDa proteins (80–82). In the majority of patients, the autoantibodies target the soluble ectodomain of BP180, LAD-1, and 20% of the sera recognize BP180 NC16A (83). The disorder can affect both children and adults. The age of onset for the adult variant shows two peaks in the teenage years and around 60 years, respectively. The childhood variant has an age onset of 4.5 years (84). A significant association between the HLA locus and LABD has been reported. In particular, the haplotypes B8, DR3 and DQ2 increase the likelihood of an early onset and are thus commonly seen in the chronic bullous disease of childhood (CBDC) variant. In both children and adults alike, the tumor necrosis factor-2 (*TNF-2*) serves as an indicator of the increased duration of the disorder, whereas *TNF-1* indicates a reduced duration and an overall better prognosis (85).

#### 1.2.6. Mucous Membrane Pemphigoid

Mucous membrane pemphigoid (MMP) is a pemphigoid disease which predominantly affects the mucous membranes. It is also known as cicatricial pemphigoid and has an incidence of 0.5–2 new cases per million/year. In contrast to other pemphigoid diseases, a common characteristic of MMP is scarring, which may cause functional limitations of the affected tissue and adds to the severity of the disease. The age of onset varies between 60–65 years of age (71–73). The target antigens are hemidesmosome proteins, such as BP180, BP230, laminin 332, α6, β4 integrin, and collagen VII (63, 73).

## 2. Omics Approaches to AIBD Diagnosis and Disease Etiology

Clinical diagnosis of AIBD relies on the combination of clinical representation, i.e., mapping of a patients' symptoms to the aforementioned phenotypic characteristics and detection of tissue-bound auto-antibodies through direct immunofluorescence (DIF) microscopy as the *de facto* standard. Although a robust and successful approach, DIF microscopy requires cryosections of perilesional biopsies and only offers limited information about the target antigen. Circulating autoantibodies can be detected via indirect immunofluorescence (IIF) microscopy, incubating tissue substrates, e.g., monkey, rabbit or human esophagus with patient serum. Procedures that offer specific information about the autoantigen are, for example, enzyme-linked immunosorbent assays (ELISA), IIF microscopy assays, and immunoblotting/-precipitation (40).

Individual molecules and genetic associations have been unraveled in the context of AIBD pathogenesis. However, AIBDs are complex diseases, with many associations and factors contributing to their etiology (cf. Table 1). As such they should be investigated in light of a systems medicine approach on different, feedback-entangled regulatory layers such as the genome, transcriptome, epigenome, proteome, or microbiome (86–88).

However, despite recent developments in next generation sequencing and OMICS technologies, AIBDs are only now becoming the focus of genetic and high-throughput data studies, and are reviewed below.

### 2.1. OMICS Studies of AIBD

Beyond the directly affected genes and proteins listed in Table 1, there are few additional studies addressing unbiased, exploratory approaches to AIBD. We searched for AIBD OMICS datasets using Gene Expression Omnibus (GEO), European Nucleotide Archive (ENA), and the NCBI databases. Additionally, we searched for microbiome studies because of growing evidence on the microbiome-immune system crosstalk (89). All identified datasets are shown in Table 2.

**Table 2:** Available AIBD microarray or next generation sequencing datasets covering more than the known marker genes.

	**Microarray RNA-seq**	**WES WGS**	**Microbiome**	**NGS Amplicon (IgVH Repertoire)**	**GWAS**
	**Pemphigus**				
PV	–	–	–	(90–93)	(94–97)
PF	(98)	–	–	(91, 92)	(94, 96, 100)
PH	–	–	–	–	–
PNP	–	–	–	–	–
AP	–	–	–	–	–
PE	–	–	–	(101)	–
	**Pemphigoid**				
BP	–	–	(102)	–	–
PG	–	–	–	–	–
EBA	–	(99)	–	–	–
ALP	–	–	–	–	–
LABD	–	–	–	–	–
MMP	(103)	–	–	–	–

*Citations refer to published sets of data. PV, Pemphigus Vulgaris; PF, Pemphigus Foliaceus; PH, Pemphigus Herpetiformis; PNP, Paraneoplastic Pemphigus; AP, IgA Pemphigus; PE, Pemphigus Erythematosus; BP, Bullous Pemphigoid; PG, Pemphigoid Gestationis; EBA, Epidermolysis Bullosa Acquisita; ALP, Anti-Laminin γ1\p200 Pemphigoid; LABD, Linear IgA-Dermatosis; MMP, Mucus Membrane Pemphigoid; WES, Whole Exome Sequencing; WGS, Whole Genome Sequencing; NGS, Next Generation Sequencing; IgVH, Immunoglobulin Variable Region Heavy Chain; GWAS, Genome wide Association Study*.

Two whole transcriptome studies are currently publicly available. In both studies, microarrays were used to quantify transcriptome differences between patients with AIBD and the controls. Malheiros et al. (98) compared gene expression in isolated CD4+ T cells from 15 PF patients to five controls (Gene Expression Omnibus ID GSE53873). A patient subgroup comparison revealed few (up to 135) differentially regulated genes related to lymphocytes, apoptosis, proliferation and antigen presentation, which may however, be due to the small cohort size and inter-patient heterogeneity. A second, unpublished study quantified gene expression profiles in 12 patients with ocular MMP and 12 controls (Gene Expression Omnibus ID GSE77361).

More recently, the immune repertoire of PV, PF, and PE patients was classified using high-throughput sequencing methods. Two studies investigated four PV patients each (90–93). Additionally, two PF patients were investigated in (91). A third dataset is available that classifies the immune repertoire of two patients with pemphigus, without providing any further details [NCBI Bioproject ID (101)].

During the last decade, genome-wide association studies (GWAS) have become a popular approach to find genetic alterations, and so-called single nucleotide polymorphisms (SNPs) linked to diseases. Such studies compare the genotypes, i.e., a selected set of about 1-2 million SNPs, of large population cohorts in search of point mutations that significantly differ in the trait of interest. So far there are about 6,000 association studies that found more than 70,000 variant-trait associations (104). Further, association studies with gene expression as the trait (eQTL studies) (105) or methylation as the trait (meQTL) have been conducted (106). More recently, GWAS data have also been used for the computation of polygenic risk scores (107). Interestingly, the number of risk loci detected by GWAS studies scales linearly with the cohort size with no sign of saturation (108). Extrapolating on this circumstance, it is most likely that the whole genome of a person contributes to the individual disease risk. In fact, recent perspectives deny the existence of a few core genes that are causing adult-onset of disease. Instead, they argue for polygenicity or an “omnigenic” gene model, in which all mutations contribute with small effect sizes (109, 110). This is due to the robustness of biological systems, which can buffer many deleterious effects such as mutations through multiple back-up mechanisms, redundancy or feedback (111–113).

A number of GWAS for PV and PF were published recently for the Jewish (97) and Han Chinese population (94–96). Besides associations within the HLA locus, all studies found one or multiple significantly associated non-HLA SNPs, however, the associated genes differed between the studies. Another study found long non-coding RNA (lncRNA) polymorphisms associated with PF (100) using SNP data from 229 cases.

Besides the availability of just a small number of datasets for AIBDs, all studies were performed on a rather limited number of cases. Even the number of patients in the GWAS publications is low (between 100 and 365) compared to genome-wide association studies performed in other autoimmune disorders in which tens of thousands of patients were investigated [for review see e.g., (114)]. The small sample sizes lead to a small number of associated variants and genes identified by the use of GWAS.

### 2.2. Novel Approaches and Technologies in AIBD Research

Recent advances in mass spectrometry technology allows the unbiased quantification of protein abundance in an unprecedented way (115). Alongside proteomics, the field of degradomics has been established, which identifies proteases together with their cleaved substrates, or “degradomes” *in vivo* (116–118). Granzyme serine proteases in particular play important roles in the context of tissue injury and repair (119, 120). Granzyme B (GzmB) is involved in both intracellular and extracellular processes in immune cell-mediated apoptosis and extracellular proteolysis, respectively, which suggests a proteolytic role of GzmB in the pathogenesis of AIBD including BP (121). GzmB accumulated in the DEJ and blister fluid of AIBD, where it cleaved key anchoring proteins in a murine model of EBA. In line with this, GzmB deficiency reduced blistering (121).

A recent field of investigation is concerned with the organisms living in a state of symbiosis on and inside the human body. Only about 43% of cells in the human body are human, while the remaining 57% are comprised of bacterial (microbiome), fungal (mycobiome) and viral (virome) communities (122). Indeed, the total number of genes of the microbiome is ten times larger compared to the roughly 22,000 human genes. Physiological structures have adapted to the interplay between resident communities and the host, e.g., the vagus nerve represents an interface between the gut-microbiome and brain (123). The gut microbiome exists synergistically with its host to shape both metabolism and immunity. Details of this inter-dependency are still largely unknown, and it is unclear whether dysbiosis is an inducing factor leading to the exacerbation of symptoms or an epiphenomenon (124). There is also little understanding of the relationship between the skin microbiome and host defense. The communities populating the stratum corneum contribute to the first defensive line against outside influences. Furthermore, a connection between host genetics and microbial composition has been shown (125).

So far, one study investigated the role of the skin microbiota and BP with a rather limited number of patients and controls [12 per group; (102)], using a variety of skin locations (five different sampling sites). Despite the small sample size and the large heterogeneity of the skin microbiome, a significantly shifted microbiome at perilesional sites was identified.

Apart from detrimental environmental influences or lifestyle choices, e.g., injuries, pathogens and drug use, it is reasonable to assume that the respective diet has an impact. In their work Fedeles et al. (126) summarized research on the influence that dietary factors exert on bullous skin disorders. Both detrimental and advantageous effects of nutritional contents are presented, i.e., associations to exacerbation or even triggering of symptoms are drawn and protective effects are pointed out as well. This linkage is of particular interest with regards to the human microbiome.

A comparable line of investigation is the effect of the mitochondria on cellular processes. As the main source of cellular energy, mitochondria are essential constituents in signaling processes, cellular metabolism as well as inflammatory responses. It has been reported that changes in the mitochondrial genome can lead to pathological conditions and are implicated in various immune diseases. In particular, the mitochondrially encoded *MT-ATP8* gene has been linked to BP susceptibility (127).

Single-cell RNA-sequencing (scRNA-seq) is another recent technology that will have a major impact on elucidating the molecular processes in AIBD. The technology allows the characterization of individual cells, leading to the discovery of new cell types and cellular states that echo the underlying heterogeneity and plasticity of the immune system. scRNA-seq opens up new possibilities to analyze the immune repertoire and its effects on immune cells while in parallel recording both the quantitative gene expression and the repertoire sequence information for both chains (α/β, light/heavy) of the receptor. This additional feature would identify subpopulations of immune cells, that drive the disease to paracrine stimulation or recruitment of further immune cells. Although recent progress in scRNA-sequencing has been achieved, there are currently only a few studies that have investigated the human epidermis. To date, there is still no approach for BP, only for psoriasis, where Cheng et al. (128) analyzed the scRNA-seq profiles of 92,889 human epidermal cells from nine normal and three inflamed skin samples. The analysis of transcriptome levels of keratinocyte subpopulations reflects classical epidermal layers, but also strongly segmented epithelial functions such as cell-cell communication, inflammation, and WNT pathway modulation. The identification of molecular fingerprints of inflammatory skin states, including the enrichment of the CD1C+CD301A+ myeloid dendritic cell population in psoriatic epidermis, provides a critical step toward elucidating epidermal diseases of development, differentiation and inflammation.

### 2.3. A Computational Approach to AIBD

Since the number of available genetic and/or OMICS datasets is limited and hitherto provides narrow insights into the disease etiology, one can ask whether it is possible to derive phenotype-specific pathways from given gene-sets, i.e., disease specific transcriptome patterns that allow patient stratification and also provide insight about the molecular processes involved. The exploratory approach of choice was a network diffusion in order to determine the impact of each gene-set on its biological neighborhood (129). It was followed by Gene Set Variation Analysis (GSVA) (130), which enables the assignment of sample-wise pathway enrichments on the basis of the calculated diffusion scores.

The inferred pathways do, however, not enable a clear separation of either phenotypes or clinical subclasses. It can be argued that the number of known associated genes is too small for an accurate inference of protein-protein interactions and related pathways, without a significant amount of noise. A closer investigation is warranted merely on account of the heterogeneity of phenotypes and the presumed multifactorial framework of disease genesis.

## 3. Summary and Outlook

Autoimmune skin blistering diseases (AIBDs) are complex diseases, mostly with a late onset in life. They are driven by both genetic and environmental factors. So far, most of the research on AIBDs has been conducted on clinical and diagnostic aspects of the diseases. In AIBDs, autoreactive antibodies are generated that target proteins involved in cell-to-cell and cell-to-matrix adhesion in the epidermis/epithelium and at the dermal-epidermal junction. Binding of those autoantibodies leads to inflammation and the loss of function of those proteins. Depending on the autoantigen, AIBDs can be divided into pemphigus and pemphigoid diseases. However, knowledge on detailed disease mechanisms leading to the development of AIBDs is still scarce.

The limited availability of OMICS data-sets in AIBDs (cf. Table 2) illustrates the long road ahead in elucidating the causes of AIBDs. Limiting factors include low prevalence and high age of onset. Those factors hinder the acquisition of sufficient suitable test-subjects and also limit perceived importance of this topic, hence lowering the impact of scientific research in this field.

In recent years, significant efforts have been made to remedy the problem of cohort size by establishing programs that advance regional, national, and international cooperation between medical facilities and research institutions. European Reference Networks (ERN) for instance are part of the Directive on Patient Rights in Cross-Border Healthcare of 2011. They are oriented toward the handling of rare disorders through the interconnection of specialists and resources. An example, with regards to AIBDs, is the “Dimethyl fumarate for the treatment of bullous pemphigoid” (DPem) research network that connects study groups from four European countries. This particular initiative is funded by the ERA-Net for Research Programmes on Rare Diseases, an initiative with the expressed purpose of facilitating cooperative research into rare disorders (131, 132). Observance of incidence and distribution of AIBDs is the objective of the Regibul Register (133).

One way to make the best use of the limited patient data is to use theoretical concepts and test the idea of core vs. peripheral genes. Initial genome-wide association studies on bullous pemphigoid have predicted HLA genes as risk factors. These genes are common to autoimmune diseases but not specific to AIBDs and might be considered as core gene candidates. Alternatively, core genes might be those that are strongly linked to the disease phenotype. Therefore, one might expect immune system and cell adhesion related genes to be critical to AIBD. In addition to identifying core genes it will be important to find tissue specific regulatory networks that mediate the effect of the core genes to the phenotype. To study these networks, it might be important to develop further *in vitro* and *in vivo* models, be it active or passive mouse models or organotypic skin models. The latter might be generated from fibroblasts and keratinocytes of AIBD patients or derived from induced pluripotent stem cells, thereby harboring a disease genome. A third approach to better understand disease etiology is the integration of environmental factors through microbiome studies. The effect of microbiome- immune cell interaction is well established but has never been studied in detail in AIBDs. First studies indicate a dysbiosis of the skin microbiome in lesional skin, a mechanistic link to increased immune reaction or decreased barrier function of the skin which still needs to be established.

In summary, an integrated OMICS approach to study skin blistering diseases, comprising genomics, transcriptomics, and proteomics together with the microbiome and novel methods such as single cell RNAseq, is highly warranted. Research in this direction has only recently begun and much work and opportunities still remain. Besides increasing the numbers for further GWAS, we advocate for high-throughput deep phenotyping and whole genome sequencing and investigation of tissue specific gene expression to characterize cellular properties associated with disease-associated genomes. Further, the use of disease models may elucidate the polygenic complexity of the diseases.

## Author Contributions

All authors reviewed the literature and collected the data, contributed to the manuscript, read, and approved it.

### Conflict of Interest

The authors declare that the research was conducted in the absence of any commercial or financial relationships that could be construed as a potential conflict of interest.
